# Profiling the inflammatory bowel diseases using genetics, serum biomarkers, and smoking information

**DOI:** 10.1016/j.isci.2023.108053

**Published:** 2023-09-26

**Authors:** Ruize Liu, Dalin Li, Talin Haritunians, Yunfeng Ruan, Mark J. Daly, Hailiang Huang, Dermot P.B. McGovern

**Affiliations:** 1 Analytic and Translational Genetics Unit, Massachusetts General Hospital, Boston, MA 02114, USA; 2 Broad Institute of MIT and Harvard, Cambridge, MA 02142, USA; 3 F. Widjaja Family Foundation Inflammatory Bowel and Immunobiology Research Institute, Cedars-Sinai Medical Center, Los Angeles, CA 90048, USA

**Keywords:** Association analysis, Diagnostic technique in health technology, Gastroenterology, Human Genetics, Smoking

## Abstract

Crohn's disease (CD) and ulcerative colitis (UC) are two etiologically related yet distinctive subtypes of the inflammatory bowel diseases (IBD). Differentiating CD from UC can be challenging using conventional clinical approaches in a subset of patients. We designed and evaluated a novel molecular-based prediction model aggregating genetics, serum biomarkers, and tobacco smoking information to assist the diagnosis of CD and UC in over 30,000 samples. A joint model combining genetics, serum biomarkers and smoking explains 46% (42–50%, 95% CI) of phenotypic variation. Despite modest overlaps with serum biomarkers, genetics makes unique contributions to distinguishing IBD subtypes. Smoking status only explains 1% (0–6%, 95% CI) of the phenotypic variance suggesting it may not be an effective biomarker. This study reveals that molecular-based models combining genetics, serum biomarkers, and smoking information could complement current diagnostic strategies and help classify patients based on biologic state rather than imperfect clinical parameters.

## Highlights

• Molecular-based models help classify patients with IBD into their clinical subtypes
• Genetics, serum biomarker and smoking status independently contribute to IBD subtypes
• Colonic CD shares more molecular signature with UC compared with small bowel CD

## Graphical abstract

**Figure:**
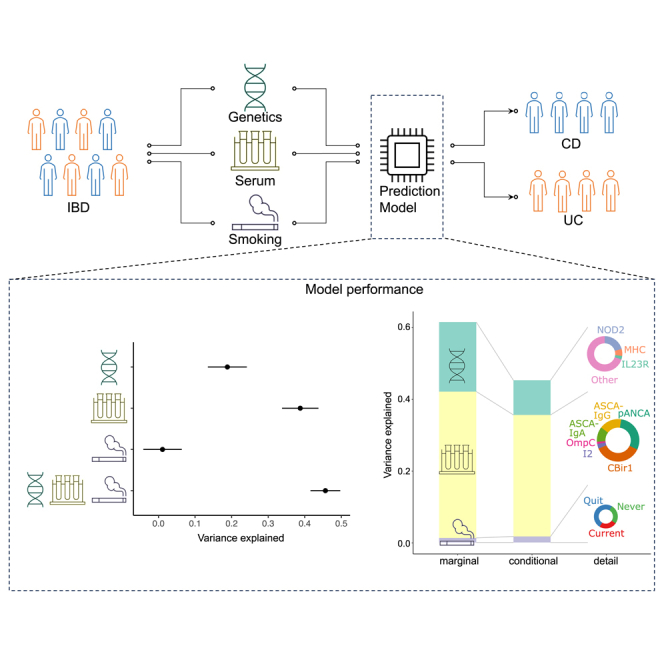

## Introduction

Inflammatory bowel diseases (IBD) are a group of chronic, debilitating disorders of the gastrointestinal (GI) tract with peak onset in adolescence and early adulthood. In 2015, 1.3% of adults (3 million) in the United States were diagnosed with IBD, a large increase from 0.9% or 2 million in 1999.^1^^,^^2^ IBD has two etiologically related subtypes: Crohn's disease (CD) and ulcerative colitis (UC). CD typically affects any part of the GI tract but is mostly localized to the ileum and is associated with full-thickness inflammation. UC, in contrast, is typically restricted to the colon and the rectum with the inflammation usually limited to the mucosal layer of colonic tissue.^3^ Clinically, the heterogeneous presentation of IBD subtypes is a major diagnostic challenge, as differentiating CD from UC can be difficult in 5–15% of patients.^4^ Diagnostic uncertainty and subsequent misclassification in IBD are associated with higher rates of complicated disease, relapse, and cancer.^5^^,^^6^^,^^7^^,^^8^^,^^9^ Furthermore, while some therapies are universally effective, others show clinical efficacy in either CD (e.g., methotrexate) or UC (e.g., mesalamine, tofacitinib) exclusively. The inability to discriminate CD from UC despite a wealth of clinical and laboratory data reflects, for some patients, an intermediate phenotype that defies conventional classification. Modern molecular techniques have paved the way for the reclassification of diseases, whereby individuals with similar molecular etiopathogenesis are likewise grouped irrespective of their “classical” diagnosis. Applying this concept to IBD might ultimately improve the prediction of clinical outcomes and response to treatment.

Several studies have attempted to differentiate CD from UC leveraging biomarkers such as microbiome,^10^ metabolites,^11^^,^^12^ laboratory markers from blood, urine and stool,^13^ the transcriptome of endoscopic biopsy tissue,^14^ and endoscopic images.^15^ For example, a microbiome-based supervised machine learning model achieved AUC >0.9 for differentiating CD and UC.^10^ Another deep learning model based on the endoscopic images had higher accuracy and less reading time than competent endoscopists for diagnosing CD and UC.^15^ More recently, metabolites in the serum have been causally connected to IBD subtypes through Mendelian Randomization^11^ and a panel of three-amino-acid metabolites was found to have a sensitivity of 88% and a specificity of 84% in discriminating patients with CD from patients with UC.^12^ However, all these studies were derived from small discovery or testing samples, raising concerns in their robustness and reproducibility. In addition, none of these predictive models incorporated genetics, which is a known determinant in IBD subtypes.^16^ In this study, leveraging a large-scale cohort that has been deeply characterized for serum antibody, smoking status and genetics, as well as IBD genetics findings from the International Inflammatory Bowel Disease Genetics Consortium, we developed and systematically evaluated a novel integrative prediction model combining all these factors.

Serum antibodies against antigens are natural biomarkers to be considered for IBD classifications and indeed have been shown to be useful in differentiating CD from UC.^17^ For example, Anti-Saccharomyces cerevisiae antibodies (ASCA) are present in 60–70% patients with CD compared with 10–15% patients with UC.^18^ In contrast, perinuclear, DNAse-I sensitive, “atypical” neutrophil cytoplasmic antibodies (*p*-ANCA) are present in 60–80% patients with UC compared with 10% patients with CD.^18^ When used in combination, serum biomarkers can be highly specific, with 85–97% specificity, in differentiating CD from patients with UC. However, they have a low sensitivity of 50–70% which impacts clinical utility.^19^^,^^20^ Additional factors to increase the sensitivity are needed to improve clinical value.

Smoking status is the only reliably associated environmental factor that has differential effects in CD and UC. Current smokers are at higher risk for CD (OR = 1.76, 95% confidence interval [CI] = 1.40–2.22) and are protected against UC (OR = 0.58, 95% CI = 0.45–0.75).^21^^,^^22^ Family studies also reported that siblings with similar genetic susceptibility tend to develop CD if smokers and UC if non-smokers.^23^ Ascertaining smoking history costs less than other biomarkers making it a natural and convenient biomarker. However, the extent to which smoking status can be used to differentiate CD from patients with UC has not been investigated reliably in a sufficiently large cohort.

Genetic factors can also be a strong predictive factor to differentiate CD from patients with UC. IBD are highly heritable with heritability of 75% for CD and 67% for UC estimated from pooled twin studies.^24^ Many genetic factors underlying IBD preferentially implicate one IBD subtype to the other. For example, a *NOD2* frameshift variant (rs5743293), which confers the strongest genetic effect on IBD risk in European ancestry populations among all known IBD associated variants, significantly increases one’s risk to CD but has almost no influence on UC risk.^25^ However, despite its strong preference toward CD, this variant, when used alone, has limited applications in classifying patients with IBD into clinically relevant subgroups because of its low population prevalence (4% in Europeans).^26^ Similarly, genetic prediction models built on a few well characterized IBD genes have been reported to have limited performance in differentiating CD from patients with UC.^27^

Recent studies have shown that polygenic risk score (PRS) can be more accurate than combined clinical risk factors currently used for population screening for diseases such as breast cancer.^28^^,^^29^ PRS aggregates the effects of genetic variants across the genome to measure the overall genetic liability to a trait or disease.^30^ Genome-wide association studies (GWASs) have reported more than 200 associations with IBD,^31^^,^^32^ potentially enabling the application of PRS in identifying individuals at high risk for IBD or differentiating IBD subtypes. Studies have shown that genetic models built to identify patients with IBD from healthy individuals achieved a maximum AUC of 0.8.^33^^,^^34^ However, while studies have investigated genetic factors underlying IBD subtypes,^16^ the extent to which genetics can be used to predict the IBD subtypes has never been studied.

Serum biomarkers, tobacco smoking status and genetics can each be valuable to inform about IBD subtypes, but they either have limited sensitivity or have not been fully evaluated. In response, we develop a prediction model combining all these factors with the goal to maximize the accuracy in differentiating CD from UC for patients with IBD. We evaluate this model using a large-scale IBD cohort to fully and robustly explore the individual and the joint contributions from these factors. We advance from earlier studies^27^ with a sample size that is several times larger, with Immuno-chip-wide immune-related regions instead of using just a few genes, and using a proper control for population structure which is essential in ensuring accurate and relevant results.

## Results

### Differentiating Crohn disease and ulcerative colitis using genetics data

We first assessed the ability of using genetics data to differentiate CD from patients with UC. To do this, we trained our genetic prediction model on the data from non-Jewish European ancestry subjects from the International Inflammatory Bowel Disease Genetics Consortium (IIBDGC, 15,987 CD and 12,613 UC, Tables 1 and S1), and evaluated its performance on the samples from Cedars-Sinai Medical Center (CEDARS, 1,947 CD and 1,100 UC, Tables 1 and S1 and STAR methods). All subjects were genotyped on ImmunoChip, an array designed to have high-density coverage in 186 loci known to be associated with autoimmune disorders.^35^ We performed 1,000 bootstrap sampling on CEDARS samples, i.e., we sampled with replacement resulting in new datasets of the same sample size, to evaluate the variance of the estimate (STAR methods and Table S2). Non-Jewish and Jewish CEDARS samples were evaluated independently.

**Table 1:** Sample characteristics for IIBDGC and CEDARS

	Non-Jewish	Jewish
**IIBDGC samples**		

IBD subtypes		
CD	15,987	1,508
Colonic	2,737	139
Small bowel	8,754	736
UC	12,613	1,115
Sex		
Male	13,117	1,434
Female	15,483	1,189
Smoking		
Current	3,919	334
Never	9,994	899
Quit	3,901	251

**CEDARS samples**		

IBD subtypes		
CD	1,947	990
Colonic	276	118
Small bowel	999	562
UC	1,100	541
Sex		
Male	1,543	820
Female	1,504	711
Smoking		
Current	416	161
Never	1,547	878
Quit	338	220
Serum biomarkers	2,907	1,459

**Figure 1:**
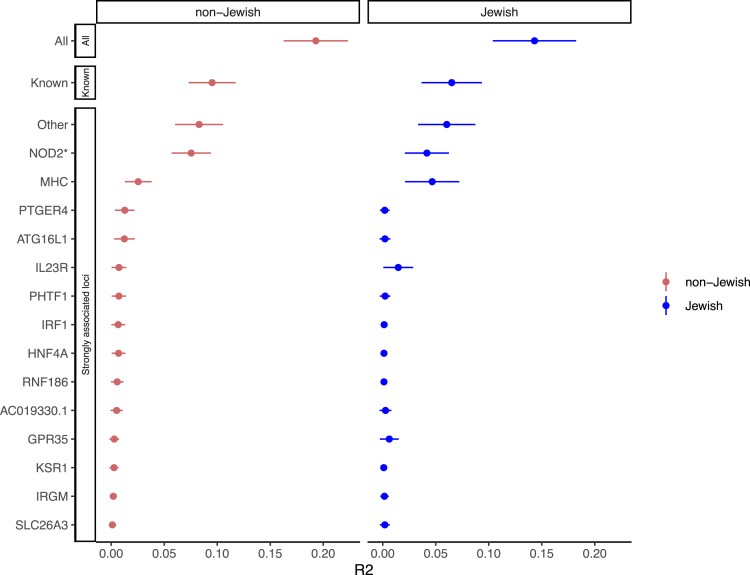
Variance explained by genome-wide or locus-based genetic prediction models p-value cut-off of 0.1 was used except for NOD2 for which the putative causal variants from fine-mapping were used (and therefore marked with “∗”). Models were tested on non-Jewish and Jewish CEDARS samples. All: the genome-wide model; Known: models using index variants from known IBD loci^38^; Other: models using genomic regions other than regions listed in the figure. Error bar: 95% confidence interval.

To build the genetic prediction model, we first performed genome-wide association analysis (GWAS) with the IBD subtypes (CD and UC) as the trait (association analysis, STAR methods and Figures S1A and S1B, Table S3. Genome-wide association test statistics using IBD subtypes (CD and UC) as the trait, related to Figure 1, Table S4. Genetic loci implicated by genome-wide significant variants in Table S3, related to Figure 1, Table S5. Test statistics in the MHC locus, using IBD subtypes (CD and UC) as the trait, related to Figure 1). The PRS for each sample was calculated using clumping and thresholding (P + T) approach, with the exception of the *NOD2* locus for which we replaced the P + T score with a score calculated from putative causal variants from fine-mapping to fully capture its genetic contribution (polygenic risk score, STAR methods). We chose P + T to calculate the PRS as it is unclear how well the Bayesian PRS methods (e.g., LDpred and PRS-CS) perform on ImmunoChip, which has very sparse coverage of the genome beyond the 186 designated high-density loci (discussion).^35^^,^^36^^,^^37^ p-value cut-off of 0.1 was used for the results with additional p-value cut-offs evaluated and found to make no significant difference (Figure S2).

We found the variance explained (STAR methods) for non-Jewish subjects was 0.193 ± 0.030 (Figure 1, error bar indicates 95% confidence interval. Same for all unless specified otherwise). For Jewish subjects the variance explained dropped to 0.143 ± 0.039, which is expected as the model was trained using non-Jewish samples (IIBDGC: non-Jewish: 15,987 CD and 12,613 UC; Jewish: 1,508 CD and 1,115 UC). We also found that known IBD loci, defined in ref. 38, make almost half of the contributions to distinguishing CD from UC with variance explained for non-Jewish and Jewish subjects 0.095 ± 0.022 and 0.065 ± 0.028, respectively (Figure 1).

Genes making the greatest contribution to the genetic model include *NOD2*, the MHC locus, *PTGER4*, *ATG16L1*, *IL23R, PHTF1, IRF1, HNF4A,* and *RNF186*, which as expected, are the top significant genetic loci from GWAS (Figure S1; Table S4). But in aggregation these top genes, defined as 13 genes mapped from the top 30 most “strongly associated loci” (many overlap with the known IBD genes described earlier), explained only half of the phenotypic variance explained by the genome-wide model (Figure 1). This observation is consistent with the polygenic genetic architecture of IBD and justifies using genome-wide data in the genetic model.

Studies have suggested that the genetic prediction accuracy can be improved by using the putative causal variants from fine-mapping instead of variants from P + T, as the former has a better “signal-to-noise” ratio. We found that by using putative causal variants from a fine-mapping study,^39^ the variance explained by *NOD2* improved from 0.055 ± 0.017 to 0.075 ± 0.018 (Figure S3) but stayed roughly the same for other genetic loci including MHC (Figure S3).^40^ Therefore, we used the putative causal variants from fine-mapping for *NOD2* and kept using the P + T variants for other loci for the final genetic model.

Our findings are unlikely to be affected by the proportions of CD and UC in the training dataset. We found that the differences in variance explained is not of statistical significance when genetic models were trained on full IIBDGC dataset with the IIBDGC CD/UC ratio of 56%:44%, CEDARS CD/UC ratio of 64%:36%, balanced CD/UC ratio of 50%:50% and so forth (Figure S4). Similarly, we observed no statistically significant differences when the CD/UC ratios in the testing dataset varied (Figure S4). The total sample size of the training data, however, is a key factor in influencing the performance of the prediction model as expected. The variance explained by the predicted model increased with the increasing sample size of training data (Figure S5).

To assess the robustness of our findings to the diagnosis noise in the training set, we trained our model on the IIBDGC data with a randomly selected proportion of CD and UC subjects flipped to each other to create a “misdiagnosis noise.” We found that training on such “noisy” samples decreased the accuracy of prediction as expected. For example, when CD and UC were flipped in an equal manner in 20% of total IIBDGC subjects, our model explained ∼75% of the original variance explained compared to the model training on the clean data (Figure S6A). However, in clinical practice, 90% of IBD subtype misdiagnoses were patients with CD wrongfully diagnosed with UC, while 10% were patients with UC wrongfully diagnosed with CD. When we simulate the CD:UC flipping in this manner (9 CD flipped to UC for every UC flipped to CD), the performance of the prediction model was not dramatically affected by the noise. Even if 50% of the training samples had their diagnosis flipped, our model still explained ∼80% of the variance explained by the model trained on the clean data (Figure S6B), likely due to the retained quality of UC diagnosis.

All analyses described above used IIBDGC samples as training and CEDARS samples as testing. These cohorts are independent and have no overlapping subjects. As a validation, we also used bootstrap to evaluate our genetic model on the IIBDGC samples. To ensure a fair comparison, we trained the genetic model using 50% IIBDGC samples and tested it on the remaining 50% IIBDGC samples and then the CEDARS samples respectively. This analysis was repeated 1,000 times. We found the variance explained was not significantly different, confirming the robustness of our results (Figure S7).

### Contribution from serum biomarkers and smoking status

**Figure 2:**
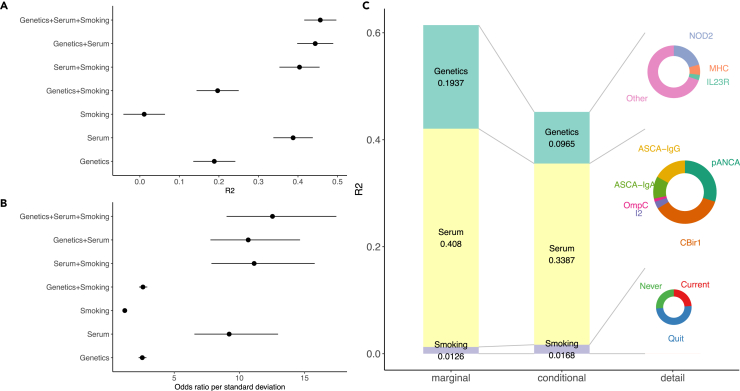
Marginal and conditional variance explained by genetics, serum biomarkers, smoking, and their joint models (A) marginal variance explained by genetics, serum biomarkers, smoking, and their combinations. (B) odds ratio (OR) per standard deviation increase of the score for genetics, serum biomarkers, smoking and their combinations. (C) marginal and conditional variance explained by genetics, serum biomarkers, smoking and each of their constituent factors. Error bar indicates 95% confidence interval.

Next, we explored the contribution of IBD-associated serum biomarkers (ASCA, ANCA, anti-CBir1, OmpC, and I2) and smoking status to differentiate CD from patients with UC. The analysis was only performed on the CEDARS samples as these are the only subjects for which the serum biomarkers and smoking status are available. We found most of the serum biomarkers were not correlated with each other (*R*^2^ < 0.1), except for ASCA-IgM and ASCA-IgG (*R*^2^ = 0.52), and I2 and OmpC (*R*^2^ = 0.39) (Table S6). We performed 1,000 replicates of 2-fold cross-validations, with 50% samples used as training and the remaining samples as testing (STAR methods). We found the serum biomarkers explain 0.388 ± 0.050 of the total phenotypic variance, and smoking status explains 0.011 ± 0.052 (Figure 2A). As expected, the model combining all the factors (genetics, serum biomarkers and smoking status, “full model”) has the best prediction accuracy, explaining 0.456 ± 0.041 of total phenotypic variance (Figures 2A and S8). The odds ratio (OR) per standard deviation increase of the full model score is 12.54 (CI = 9.02–17.44), meaning on average, individuals ranked in the top quantiles as CD have odds of CD hundreds of times greater compared with individuals ranked in the bottom quantiles (Figure 2B).

We then investigated how each factor contributes to the prediction accuracy conditional on other factors. We found the conditional contributions from genetics and serum biomarkers shrink from their marginal contributions, suggesting overlapping contributions across these factors: the variance explained by genetics reduced from 0.19 to 0.09, and serum biomarkers from 0.41 to 0.34 (Figure 2C). In the conditional analysis, serum biomarkers accounted for a greater proportion of phenotypic variance than genetics, indicating that they may have captured a sizable amount of environmental factors contributing to IBD subtypes that were not explained by genetic factors. The conditional contribution from genetics is, however, still significant, suggesting genetics contributes additional information that is not explained by the serum biomarkers. The contribution from smoking status stayed similar despite being small (variance explained from 0.012 to 0.016), suggesting its independent role in the disease manifestation (Figure 2C).

We found almost 21% of the variance explained by the genetic model was accounted for by *NOD2*, and 6% was accounted for by the MHC locus (Figure 2C). The remaining genomic regions accounted for ∼70% of the variance explained, consistent with the polygenic genetic architecture of IBD. Among serum biomarkers, CBir1 makes the largest contribution (36%) and the majority (95%) of the variance explained was accounted for by the top four serum biomarkers: CBir1, ANCA, ASCA-IgG, and ASCA-IgA.

To investigate the causal relationship between subtypes of IBD and serum biomarkers, we performed a two-sample Mendelian Randomization (MR) analysis (STAR methods). The causal effects of IBD subtypes on serum biomarkers were assessed using the inverse variance weighted MR method (IVW) and MR Egger^41^ (Figure S9; Table S7). MR Egger is an MR method with correction for horizontal pleiotropy. We found subtypes of IBD have significant causal effects on serum biomarkers using IVW, including ANCA (p = 3.02e-5), CBir (p = 5.98e-16), ASCA-IgA (p = 2.74e-10), and ASCA-IgG (p = 1.97e-9) (Bonferroni corrected p-value threshold: 0.0083 = 0.05/6). However, MR Egger failed to replicate any of these findings at Bonferroni corrected p-value threshold (0.0083 = 0.05/6, Table S7) despite that neither significant heterogeneity (heterogeneity Q test) nor pleiotropy (MR-Egger intercept close to zero) was detected (with Bonferroni correction, Table S7). Taken together, our data does not unambiguously support that the IBD subtype has a causal effect on serum biomarkers. We were not able to evaluate the causal effects of serum biomarkers on IBD subtypes because no genetic variant was significantly associated with serum biomarkers to be used as an instrumental variable.

### Disease location and subtypes

**Figure 3:**
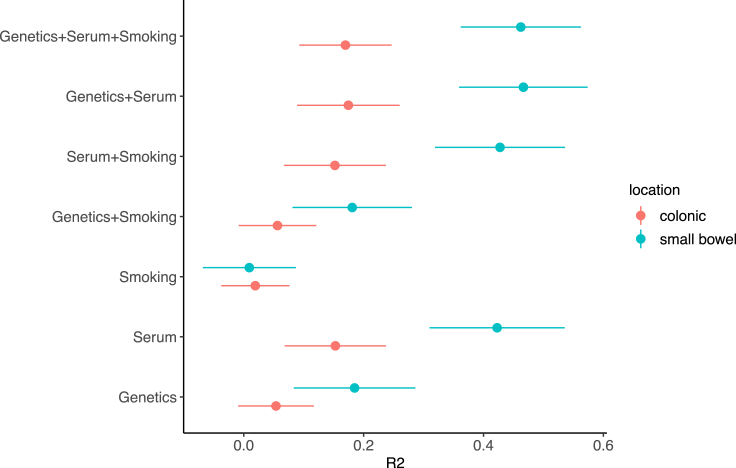
Variance explained by models with serum biomarkers, smoking and their combinations tested on colonic CD vs. UC and small bowel CD vs. UC Error bar: 95% confidence interval.

Patients with CD can be classified based on their disease location into those with exclusively colonic CD (L2) and those who have small bowel involvement (L1 and L3).^42^ CD with colonic presentation can be more challenging to be differentiated from patients with UC. In addition, different CD locations are associated with differences in the disease progression and complications,^42^ suggesting an underlying molecular basis. Indeed, using the CEDARS subjects with 1000 times 2-fold cross-validation, we found our full model explains 0.462 ± 0.100 of total variance for small bowel CD vs. UC, compared with 0.170 ± 0.077 for colonic CD versus UC (AUC: 0.710 ± 0.047; Figure 3; sensitivity: 0.708 ± 0.191, specificity: 0.651 ± 0.196, Youden index method^43^). This reduced performance is observed for models based on genetics and the serum biomarkers individually (Figure 3) and the genetic model was validated using the IIBDGC subjects (Figure S10). Importantly, this finding suggests colonic CD shares more molecular signature with UC compared with small bowel CD.

To further investigate the genetic basis of disease location for patients with CD, we performed a GWAS on the location of CD (colonic CD vs. small bowel CD) as a trait. Given the sample size limitation in Jewish samples, we only analyzed non-Jewish samples. We found 3 loci were significantly associated with the location of CD (p-value < 5e-8, Figure S11). Two of these loci, *NOD2* and MHC are consistent with previous studies that these regions are associated with IBD disease location.^16^ The third locus, *PLCH2*, contains a missense variant, rs41315664 (p-value = 2.15e-10). Further, we found models only including the *NOD2* fine-mapped variants or MHC explained 1.7% or 1.7% of the CD location variance, respectively. While the genome-wide model explained 4.3% of the CD location variance (Figure S12), suggesting the polygenic basis of CD disease location. When we expanded the model by including the serum biomarkers and smoking, this explained 9.7% of CD location variance, confirming the different molecular basis between colonic CD and small bowel CD (Figure S13).

It is worth noting that we found a significant association between smoking behavior and the location of CD (Chi-Square test p-value <2.2e-16): 31.9% of Ex-smoking (Quit) patients with CD and 25.4% of Never smoking (Never) patients with CD had colonic CD. In comparison, only 18.3% of current smoking (Current) patients with CD were colonic CD (Table S8). As recent studies suggested genetic risk for IBD can be modified by smoking,^44^ we performed a genome-wide interaction analysis with the location of CD as a trait to test for the interaction between smoking behavior and genotypes of variants. The analysis did not yield any genome-wide significant finding (p-value < 5e-8).

### The clinical application with the combined model

We evaluated the ability of the full model (with genetics, serum biomarkers and smoking status) to accurately predict the 23 “class switch” individuals in CEDARS. These individuals received an initial diagnosis of UC and underwent colectomy but their diagnosis was flipped to CD later in their disease course. Unfortunately, the full model was not able to significantly distinguish these individuals from patients with UC, probably, at least in part, because they are colonic CD subtype and therefore, there is a much more limited prediction accuracy. We also reviewed the charts of the 40 IBD unclassified patients in IIBDGC whose predicted diagnosis are in the top 5% quantiles (20 predicted CD and 20 predicted UC). Unfortunately, our predictions do not significantly align with the re-assessed diagnosis. These findings likely also reflect the limited statistical power of these analyses and further work on larger cohorts may be more informative.

## Discussion

We investigated prediction models using genetics, serum biomarkers and smoking status to facilitate the diagnosis of patients with CD and UC. Samples from IIBDGC and CEDARS were used to train and validate these models. To our knowledge, this is the first study to investigate the contribution of all these factors on the genome-wide scale using a large-scale cohort. Our results demonstrated that a model combining the molecular and environmental information may complement current diagnostic strategies and help classify patients based on biologic state rather than imperfect clinical parameters.

We found serum biomarkers make the greatest contribution perhaps as it is more closely connected to disease status. Studies have reported anti-CBir1 uniquely associated with CD and ANCA predominantly associated with UC.^45^^,^^46^ Our finding is also consistent with a previous report that the combination of anti-CBir1 and ANCA has good power of distinguishing UC from CD.^45^ However, the two-sample Mendelian randomization failed to establish a reliable causal relationship between IBD subtypes and serum biomarkers, leaving uncertainty about whether the prediction is driven by causality or association. We also demonstrated that although the differential UC/CD effect of smoking is well-established in our study, it only explains a small proportion of the phenotypic variance. This suggests that smoking is not fit to be a good biomarker, by itself, to differentiate CD from patients with UC. A Mendelian Randomization also suggested smoking may not causally influence the risk of IBD.^47^ We found genetics, even when conditioned on serum biomarkers, makes significant contributions to the prediction accuracy suggesting the human genome hosts IBD subtype relevant information that is not fully captured by the serum biomarkers. As sample sizes for genetic studies continue to increase, more IBD-associated genetic variants will be discovered and can be expected to make bigger contributions to disease classification in the future.

In addition to increasing the sample size, one strategy to improve the accuracy of the genetic model is to use the fine-mapped putatively causal variants as they better capture the genuine causal genetic effects. We have demonstrated the benefit of this for the *NOD2* gene but the application to other genetic loci yields minimal improvements (Figure S3). For example, for the MHC locus, despite using the well fine-mapped variants from a recent study,^40^ we failed to observe a performance better than the naive P + T method. The reasons could be: 1) variants in *NOD2* have shown strong preference to CD versus UC, while other loci including MHC could play roles in both CD and UC with a weaker preference; 2) *NOD2* is among the most powered IBD associated loci, allowing a comprehensive set of the causal variants to be fine-mapped (ten causal variants in this study). In other loci with no sufficient power to isolate a comprehensive list of causal variants, P + T captures additional signals at sub-thresholds which were missed in fine-mapping and thus provides a better prediction accuracy. Additionally, the long-range LD structure in MHC could also make fine-mapping more challenging, reducing the gain from using the putative causal variants from fine-mapping.

Rare variants may account for a large proportion of missing heritability for human complex traits such as height and BMI.^48^ However, the extent to which they contribute to IBD risk and how they improve the polygenic risk prediction remains to be investigated. The recent CD exome sequencing study (WES) with over 100,000 subjects offered an initial evaluation.^49^ We found that only 0.3% CD heritability was explained through new coding variants identified in that study (the largest to date), while known coding variants, captured through ImmunoChip as in our study, explained 5.1% CD heritability. This suggests that, at the current stage, including additional rare coding variants from WES may not help to increase the variance explained in IBD risk prediction.

Gut microbiota is an environmental factor that plays an important role in the pathogenesis of IBD.^50^ Recent studies have shown that the abundance of gut microbiota was associated with subtypes of IBD. Moreover, a gut microbial metabolic model suggested that CD and UC have different microbial metabolic fluxes/pathways.^51^ A recent study has shown that a gut microbiome-based supervised machine learning model could be used for differentiating CD and UC and achieved AUC > 0.9.^10^ In addition, many IBD-associated genetic variants implicate genes with immune function or influence host-microbiome interactions, such as *NOD2* and *ATG16L1.*^50^ This indicates that, while we were unable to incorporate microbiome data in this study, microbiome data may provide additional information and adding microbiome data to the model may improve the prediction accuracy on subtypes of IBD.

While only the naive P + T method was used for PRS, we found our conclusion is unlikely to change if we use more sophisticated approaches. Repeating the analysis using PRScie-2^52^ showed almost the same results, which is expected as the core of PRSice-2 is P + T (Figure S14). Further, results from using PRS-CS,^37^ a Bayesian PRS method, and multi-layer perceptron, a neural network (NN) model, showed almost no difference from the P + T results (Figure S14). We hypothesize that most genetic loci contribute to IBD disease risk through an additive fashion, which can be well captured in a linear model. The neural network additionally includes non-linear terms but it requires a big training sample size to fit those non-linear genetic effects. At the sample size of our study, the neural network may not be able to effectively train its non-linear effects and thus had limited improvements in the prediction accuracy. Our findings are consistent with a previous CD prediction study that neural networks provided similar performance as the logistic regression model.^34^

A major concern about PRS is that it has limited portability across populations.^53^^,^^54^ We confirmed that as our model was trained on non-Jewish subjects, the model’s performance decreased when tested on Jewish subjects, suggesting the PRS model may not be fully transferable to subjects of ancestries different from the ancestry of training data. This is consistent with previous studies that genetic risk prediction model has decreased cross-ancestry and within European ancestries prediction accuracy,^53^^,^^55^ an issue that needs to be addressed to accelerate the equitable deployment of PRS in clinical setting and maximize its healthcare potential.

Colonic inflammation occurs in both CD and UC. Previous work has shown that ileal CD, colonic CD and UC can be distinguished by their genetic risk score, with colonic CD showing a genetic risk score between ileal CD and UC.^16^ Our study confirmed that colonic CD is more similar to UC than non-colonic CD in molecular profile. These findings are important as the most effective IBD therapies currently available appear less effective in small bowel compared to colonic CD.^42^ Understanding the different molecular etiologies of small bowel and colonic inflammation will be important to develop more personalized strategies to treat small bowel disease which is associated with more complicated diseases.^56^ Furthermore, it has been suggested that historical classification approaches to classifying disease location in CD should evolve to reflect the differences in small bowel and colonic CD and our data strongly support this.^42^

A previous study reported that a model combining serology, genetics, and inflammation markers discriminated CD and UC with AUC 0.93 ± 0.04 using 373 patient samples.^27^ The combined model was an improvement over the serological biomarker model, which had an AUC 0.78 ± 0.06. Our findings are consistent with the previous study, as we also found that the combined model that aggregated genetics, serum biomarkers, and tobacco smoking information performed significantly better than the model that contained only one of those factors. The AUC of our combined model is 0.856 ± 0.0154 which is smaller than the number reported in the previous study likely due to their inclusion of inflammation markers. The difference could also be driven by the proportion of patients with colonic CD in the studies. The AUC using serum biomarkers only was 0.78 ± 0.06 (95% CI) in the previous study and 0.834 ± 0.018 (95% CI) in our study, suggesting the contribution of serum biomarkers to distinguishing CD from UC in the two studies are roughly the same despite a slight difference in the choice of biomarkers (previous study included anti-A4FlaX and anti-Fla2 rather than I2 in our study). We improved the previous study in the following: (1) Sample size. Our model was trained and tested with 5x sample size of the previous study. The sample size and the more diverse range of European ancestries included ensured the transferability of our findings. (2) We used Immuno-chip-wide PRS instead of 4 genetic markers which more accurately captures the genetic architecture of IBD. As a result, the genetic component captured 0.193 ± 0.030 of IBD subtype variance. (3) We investigated the contributions of each factor alone and jointly for subtypes of IBD which were not performed in the previous study. We found that these factors contribute to IBD subtypes in both independent and overlapping ways. We used variance explained (R^2^) to measure the performance rather than AUC because it provides additional control over the population structure and allows us to evaluate the marginal and conditional contribution from each factor. (4) We stratified patients with CD based on the disease location and found that the performance of CD/UC classification using the current model had better performance for small bowel CD than patients with colonic CD. (5) We performed MR analysis to explore the causal relationship between serum biomarkers and IBD subtypes. We didn’t find that the IBD subtype has a causal effect on serum biomarkers.

While this study is well powered with the large-scale genetics samples, the sample size is limited by the number of samples that also have serology and disease location. Additionally, this study did not include microbiome data. This prevents us from more accurately evaluating the joint model, especially its clinical utility, on a large scale. Nevertheless, our study has clearly demonstrated that a molecular and environmental factor-based model combining genetics, serum biomarkers, and smoking information could potentially complement current diagnostic strategies and help classify patients based on biologic state rather than imperfect clinical parameters.

### Limitations of the study

The limitations of our study include: (1) The complex and multifactorial nature of IBD limits the accuracy of our model. Therefore, our model cannot be fully used in the clinical setting at this time. (2) We only focused on predicting the subtypes of IBD and two disease locations of CD. We were unable to comprehensively evaluate other important factors, such as the age of onset, disease severity, complication, disease course, response to therapy, amount of smoking, and gut microbiota, due to limitations in the phenotype and biomarker data of our samples in either IIBDGC or CEDARS. (3) Although we have data on disease extent and behavior, these factors have been evaluated and are known to have smaller heritability.^16^ When combined with the reduced sample size (due to the restriction to less than 10% of subjects with serum biomarker data), we did not have the statistical power to properly model these factors. While our study has a few limitations, we believe it is valuable in providing the initial evaluation of the contribution of genetics, serum biomarkers, and smoking information to CD/UC classification in a sizable cohort.

## STAR★Methods

### Key resources table

**Table:** 

REAGENT or RESOURCE	SOURCE	IDENTIFIER
**Software and algorithms**		

plink	Chang et al.^61^	https://www.cog-genomics.org/plink/
Beagle	Browning et al.^58^	http://faculty.washington.edu/browning/beagle/beagle.html
Admixture	Alexander et al.^62^	https://dalexander.github.io/admixture/
rcompanion	Salvatore Mangiafico^64^	https://cran.r-project.org/web/packages/rcompanion/index.html
PRS-CS	Ge et al.^37^	https://github.com/getian107/PRScs
scikit-learn	Pedregosa et al.^65^	https://scikit-learn.org/
TwoSampleMR	Hemani et al.^66^	https://mrcieu.github.io/TwoSampleMR/

### Resource availability

#### Lead contact

Further information and request for resources should be directed to and will be fulfilled by the lead contact, Hailiang Huang (hhuang@broadinstitute.org).

#### Materials availability

This study did not generate new unique reagents.

### Experimental model and study participant details

#### Participants

##### IIBDGC

Individual-level genotypes were obtained from the International Inflammatory Bowel Disease Genetics Consortium (IIBDGC). The detail of recruitment and recruitment sites were described in^32^ and Table S1 from.^16^ Briefly, the recruitment of patients was performed in 15 countries in Europe, North America and Oceania. The case ascertainment approach for this study was based on the European Evidence-based Consensus or the ECCO-ESGAR Guideline for Diagnostic Assessment in IBD. Diagnosis of IBD was based on accepted radiological, endoscopic and histopathological evaluation. All included patients fulfilled clinical criteria for IBD. For this study, we excluded samples from CEDARS in the IIBDGC samples, for a total of 17,495 CD and 13,728 UC individuals (Table 1). All samples have undergone quality control (QC) as described previously.^39^

##### CEDARS

Participants in CEDARS were recruited at the IBD and pediatric IBD Centers at Cedars-Sinai Medical Center, Los Angeles, USA. The details of recruitment and recruitment sites were described in.^57^ The case ascertainment approach for this study was based on the European Evidence-based Consensus or the ECCO-ESGAR Guideline for Diagnostic Assessment in IBD. Diagnosis of IBD was based on accepted radiological, endoscopic and histopathological evaluation. All included patients fulfilled clinical criteria for IBD. After filtering out non-European ancestry and IBD unclassified participants, there are 2,937 CD and 1,641 UC subjects available (Table 1). Individual-level genotypes, serum biomarkers, and smoking status are available in CEDARS. QC for CEDARS samples was described in a previous study.^57^

All participants in this study are of European ancestry, with 11.6% of Jewish ancestry. 47.24% of participants are male and 52.76% are female. The mean age at diagnosis of participants was 31 years, with a standard deviation of 15 years. All research-related activities were approved by the Mass General Brigham Institutional Review Board and/or the Cedars-Sinai Medical Center Institutional Review Board.

### Method details

#### Variants

All analyses in this study were performed on a set of variants that are shared by both IIBDGC and CEDARS samples post-QC (129,199 variants). Due to the high density design of the ImmunoChip, we did not perform imputation except for the MHC locus, for which we imputed variants within the class Ⅰ and class Ⅱ HLA genes at the level of classical HLA alleles and 4,282 amino acids in IIBDGC and CEDARS samples using Beagle^58^ (version 5) and the T1DGC MHC reference panel.^59^ 8,312 variants and HLA alleles with imputation quality >0.6 in both samples, plus 936 variants in the ImmunoChip design were used for further analysis.

#### Smoking status

Tobacco smoking was considered as a categorical variable, with three levels: (a) IBD patients who have never smoked; (b) previous smokers who quit before their IBD diagnosis; and (c) patients who were smokers at the time of IBD diagnosis.

#### Serum biomarker ascertainment

Serum immune responses were analyzed by ELISA on the CEDARS cohort as previously described^57^^,^^60^ including: anti-Saccharomyces Cerevisiae antibodies (ASCA IgG and IgA), perinuclear anti-nuclear cytoplasmic antibody (pANCA), anti-flagellin (anti-CBir1), anti-outer membrane porin C (anti-OmpC) and anti-Pseudomonas fluorescens-associated sequence I2 (anti-I2). Serum samples were typically obtained at study enrollment and consent in the Cedars-Sinai IBD Centers. All assays were performed blindly without knowledge of patient clinical characteristics.

### Quantification and statistical analysis

#### Principal component analysis and ancestry

Principal components (PC) for IIBDGC samples were taken from a previous study.^39^ For the CEDARS samples, we used the following steps to calculate the PC: 1) we removed variants within the MHC region, with MAF <0.05, with a call rate <0.99, or in violation of Hardy–Weinberg equilibrium with p-value < 1e-5. This strict quality filter allows us to ensure the top PCs capture the population structure rather than genotyping artifacts. 2) we LD pruned variants with pairwise *R*^*2*^ = 0.1, window size = 100 variants and step size = 5 variants; this was repeated three times to address the complex LD structure. 3) after pruning, 14,963 variants were used for PC analysis by PLINK.^61^

Jewish ethnicity was estimated using Admixture^62^ and the Human Genome Diversity Project^63^ and a local Jewish non-IBD study as reference samples. Individuals with >75% Jewish components were classified as Jewish.

#### Association analysis

We performed genome-wide association analysis on 15,987 CD and 12,613 UC samples from IIBDGC. We excluded projected Jewish samples and CEDARS samples in the association analysis, and only retained variants that are shared with the post-QC CEDARS samples as discussed in Sample characteristics. We performed logistic regression on variants having minor allele frequency >0.5% (114,146 variants) with the top ten PCs as covariates using PLINK. 1,944 variants were associated with IBD subtypes (CD and UC) at genome-wide significance (Figure S1A; Table S1), all of which implicate IBD loci that have been previously reported (Table S2). In the MHC locus, we additionally included the imputed HLA variants (Sample characteristics) for the maximum genomic coverage (Figure S1B; Table S3). There were 8,312 imputed variants/HLA alleles and 936 variants from ImmunoChip design included in the MHC locus, among which 2,350 variants were significantly associated with IBD subtypes.

#### Polygenic risk score

We used the pruning and thresholding (P + T) approach to calculate the PRS for study subjects. We used the clumping function in PLINK and the in-sample LD to clump genome-wide variants into independent association signals. For non-MHC regions, the clumping was performed with a radius of 250Kbp and pairwise *R*^2^ > 0.2. For the MHC region, we performed clumping 3 times with a radius of 5Mb (covering the full MHC region) and pairwise *R*^2^ > 0.1 for the first time and pairwise *R*^2^ > 0.05 for the next two times (For a total of three times). p-value thresholds were set to 0.1, 0.01, and 0.001, leading to 5,502, 1,398, and 495 clumps for non-MHC regions and 88, 65, 54 for MHC regions, respectively.

For each individual, the polygenic risk score (PRS) was calculated as Σlog(OR)∗G, in which OR is the odds ratio from the association analysis for the clumped indexed variants and G is the genotype dosage. We included all post-clumping and post-thresholding variants as mentioned above.

To calculate the score for a specific gene (e.g., *ATG16L1* and *IRF1*), we added a flanking region 300kbp up- and down-stream. The score for *NOD2* was calculated using the putatively causal variants from an IBD fine-mapping study.^39^

#### Variance explained

The variance referred to the variation of CD/UC diagnosis within IBD patients. We used logistic regression to calculate the variance explained by factors of interest. The subtypes (CD or UC) were treated as the response variable. PRS, serum biomarkers, and/or smoking status were treated as explanatory variables. We calculated the baseline model using the first ten PCs with the intercept, and the alternative model with the factors of interest (PRS, serum biomarkers and/or smoking) added to the baseline model. We then computed the nagelkerke pseudo R^2^ by comparing the alternate model with the baseline model.^64^

#### Neural network

We built the multilayer perceptron neural network models including variants after clumping p-value < 0.1, 0.01, and 0.001. We performed a grid search to determine the best model hyperparameter: the number of hidden layers (2 and 3), number of nodes per layer (100, 200, and, 500), L2 regularization alpha (1, 0.5, 0.1, 0.05, 0.01, 0.005, 0.001), the exponential decay rate for estimates of first-moment vector in adam solver (0.1, 0.3, 0.5, 0.7, 0.9), the exponential decay rate for estimates of second-moment vector (0.1, 0.3, 0.5, 0.7, 0.9) in adam solver, and activation function (logistic, relu, and tanh). The performance of the models was measured by the average AUC of 5-fold cross-validation in IIBDGC by using scikit-learn^65^ (https://scikit-learn.org/). Then the best performing model was then trained with full IIBDGC data and tested with CEDARS. Bootstrap was used to evaluate the variance of the AUC.

#### Mendelian randomization

We performed bidirectional two-sample Mendelian Randomization to test for the causal relationship between subtypes of IBD and serum biomarkers. To investigate the causal effect of IBD subtypes on serum biomarkers, we use as exposure the summary statistics from IBD subtype GWAS using IIBDGC subjects; and as outcome the summary statistics from GWAS using rank-based inverse normal transformed serum biomarker measurements as traits in CEDARS samples. To select the unrelated variants as genetic instrumental variables for subtypes of IBD and serum biomarkers, we 1) extracted variants with genome-wide significance (p < 5e-8); 2) performed clumping with *R*^2^ < 0.01 and window size = 500kb on the other region; 3) removed variants from *NOD2* and MHC, as a conservative measure, due to their potentially strong horizontal pleiotropy. For MR analysis with more than one instrumental variable, we used both inverse variance weighted fixed-effect regression (IVW) and MR-Egger regression (TwoSampleMR^66^); for MR analysis with a single genetic instrumental variable, we used the Wald ration test (TwoSampleMR). We perform Cochran’s Q test of IVW and MR-Egger for heterogeneity analysis and intercept test of MR-Egger regression for horizontal pleiotropy analysis. The causal effects of serum biomarkers on subtypes of IBD could not be tested, as there is no variant significantly associated with serum biomarkers to be used as instrument variables after excluding *NOD2* and MHC.

## Data Availability

• Genomics data that support the findings of this study are available on request from the International IBD Genomics Consortium. Serum biomarkers and smoking status data are available upon request from D.M. (Dermot.McGovern@cshs.org). Variants weight for genetic model is in https://personal.broadinstitute.org/hhuang/public/CD_UC/. • This paper does not report original code. • Any additional information required to reanalyze the data reported in this paper is available from the lead contact upon request.

## Supplemental information

Document S1. Figures S1–S14

Table S1. Demographics and clinical characteristics, related to Table 1

Table S2. Study design, related to STAR Methods

Table S3. Genome-wide association test statistics using IBD subtypes (CD and UC) as the trait, related to Figure 1

Table S4. Genetic loci implicated by genome-wide significant variants in Table S3, related to Figure 1

Table S5. Test statistics in the MHC locus, using IBD subtypes (CD and UC) as the trait, related to Figure 1

Table S6. Squared correlation coefficient matrix across serum biomarkers, related to Figure 2

Table S7. Mendelian randomization results between subtypes of IBD and serum biomarkers, related to Figure 2

Table S8. Number of CD patient stratified by CD location and smoking behavior in IIBDGC, related to Figure 3
