# Supplementary material for: Profiling the inflammatory bowel diseases using genetics, serum biomarkers, and smoking information
Source: iScience. 2023 Sep 26;26(10):108053. doi: 10.1016/j.isci.2023.108053 (PMC10568094; doi:10.1016/j.isci.2023.108053)
Supplement: Document S1. Figures S1–S14 [file mmc1.pdf]

iScience, Volume *26*

Supplemental information

Profiling the inflammatory bowel diseases using genetics, serum biomarkers, and smoking information

Ruize Liu, Dalin Li, Talin Haritunians, Yunfeng Ruan, Mark J. Daly, Hailiang Huang, and Dermot P.B. McGovern

Supplementary Figures

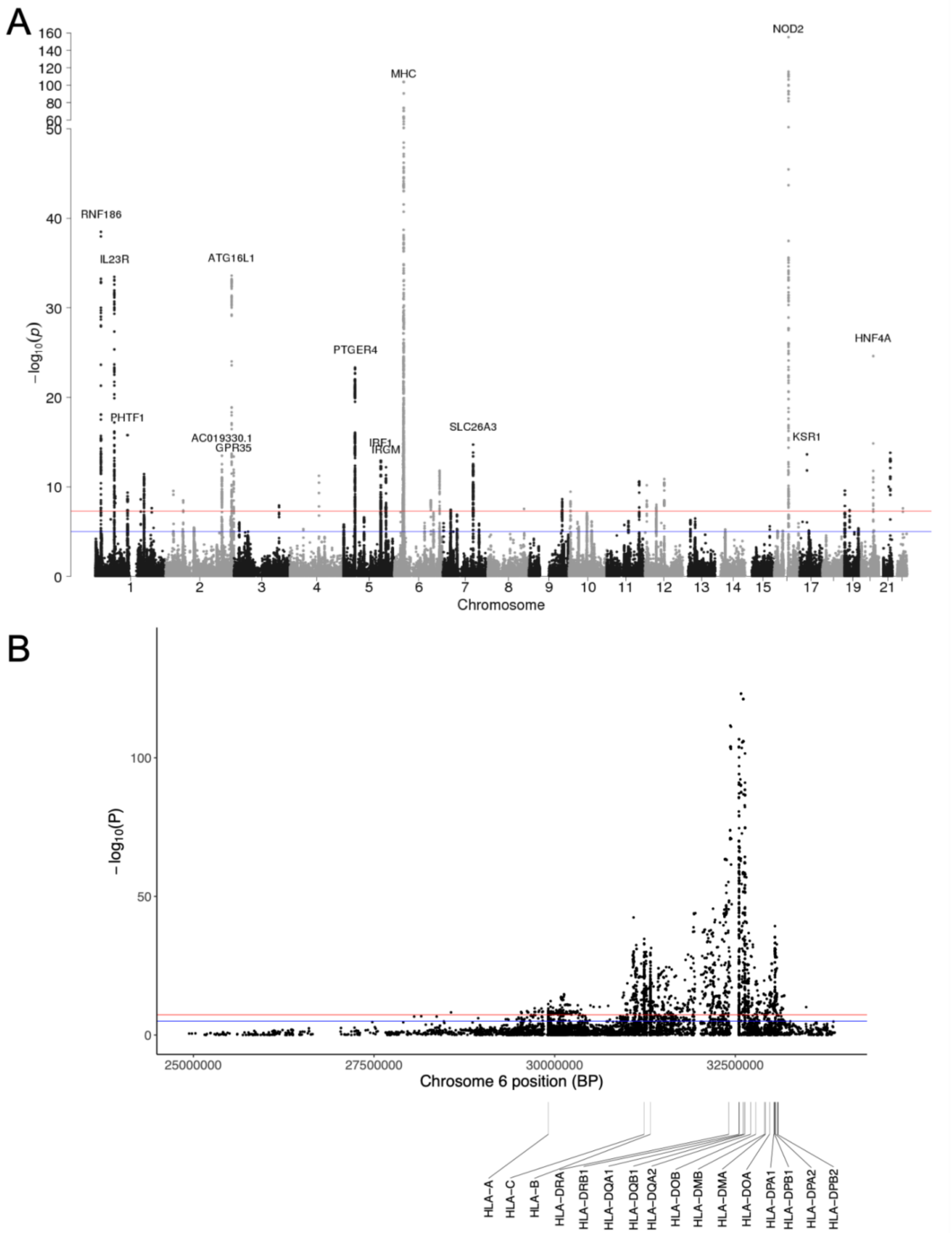

Figure S1. Genetic association for CD versus UC using IIBDGC non-Jewish samples, Related to Figure 1. (A) Manhattan plot with top associations annotated with a gene within ± 300kb. (B) Regional association plot for the MHC locus post imputation. Red line: Genome-wide significance threshold at $P=5\times10^{-8}$. Blue line: suggestive significance threshold at $P=10^{-5}$.

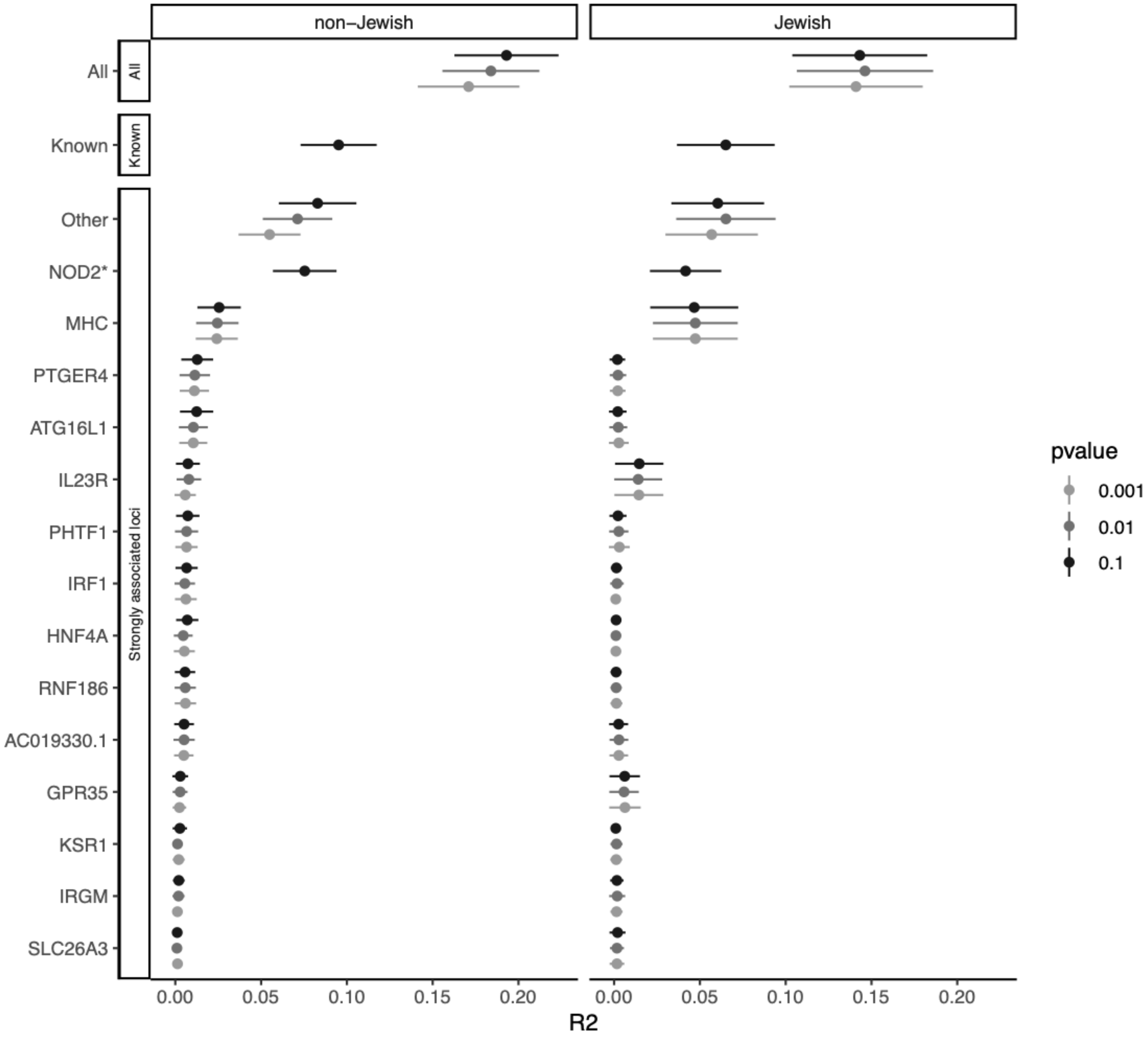

Figure S2. Variance explained by genome-wide or locus-based genetic prediction models, Related to Figure 1. *P*-value cut-off of 0.1, 0.01, and 0.001 were used respectively to build the models except for *NOD2* for which the putative causal variants from fine-mapping were used (*). The models were tested on non-Jewish and Jewish CEDARS samples. All: the genome-wide model; Known: models using index variants from known IBD loci [38]. Other: model using genomic regions other than regions listed in the figure. Error bar: 95% confidence interval.

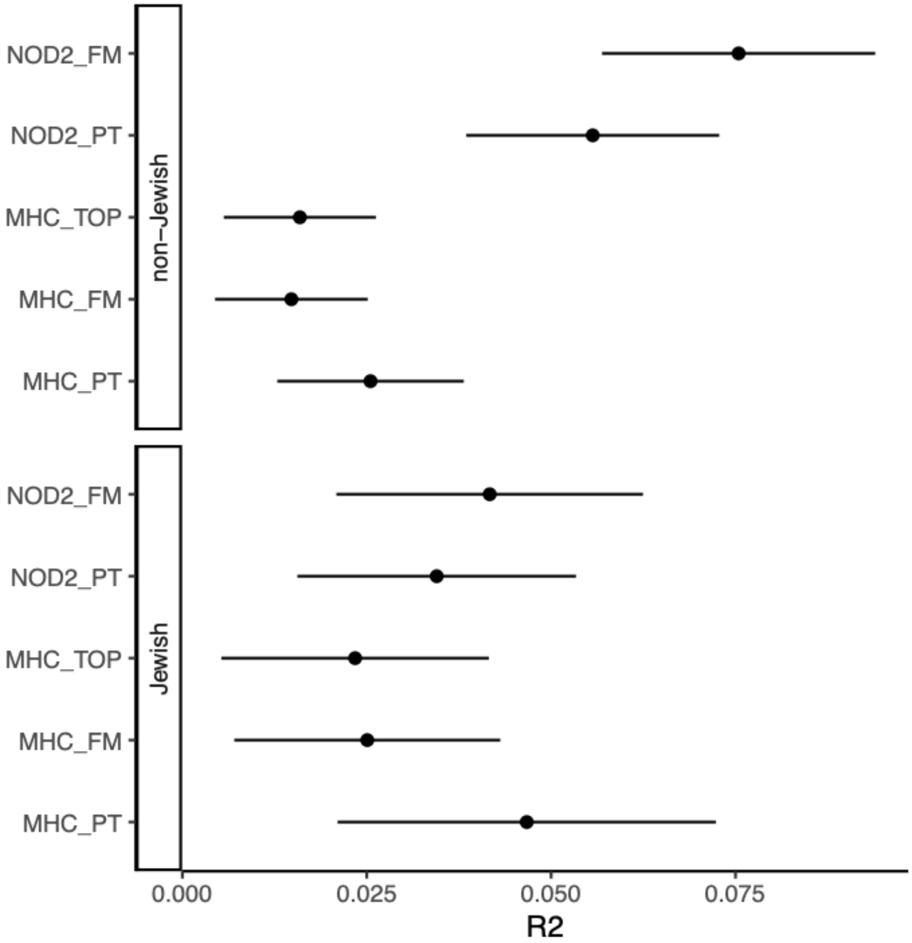

Figure S3. Variance explained by the *NOD2* and MHC loci using different genetic models, Related to Figure 1. The genetic models were tested on non-Jewish and Jewish CEDARS samples respectively. NOD2_PT: genetic model using the P+T variants (*P*-value cut-off at 0.1) for *NOD2*; NOD2_FM: genetic model using the putative causal variants from fine-mapping the *NOD2* locus, MHC_TOP: genetic model using the most significant variant from the MHC locus; MHC_PT: genetic model using the P+T variants (*P*-value cut-off at 0.1) for the MHC locus; MHC_FM: genetic model using the putative causal variants from fine-mapping the MHC locus. Error bar: 95% confidence interval.

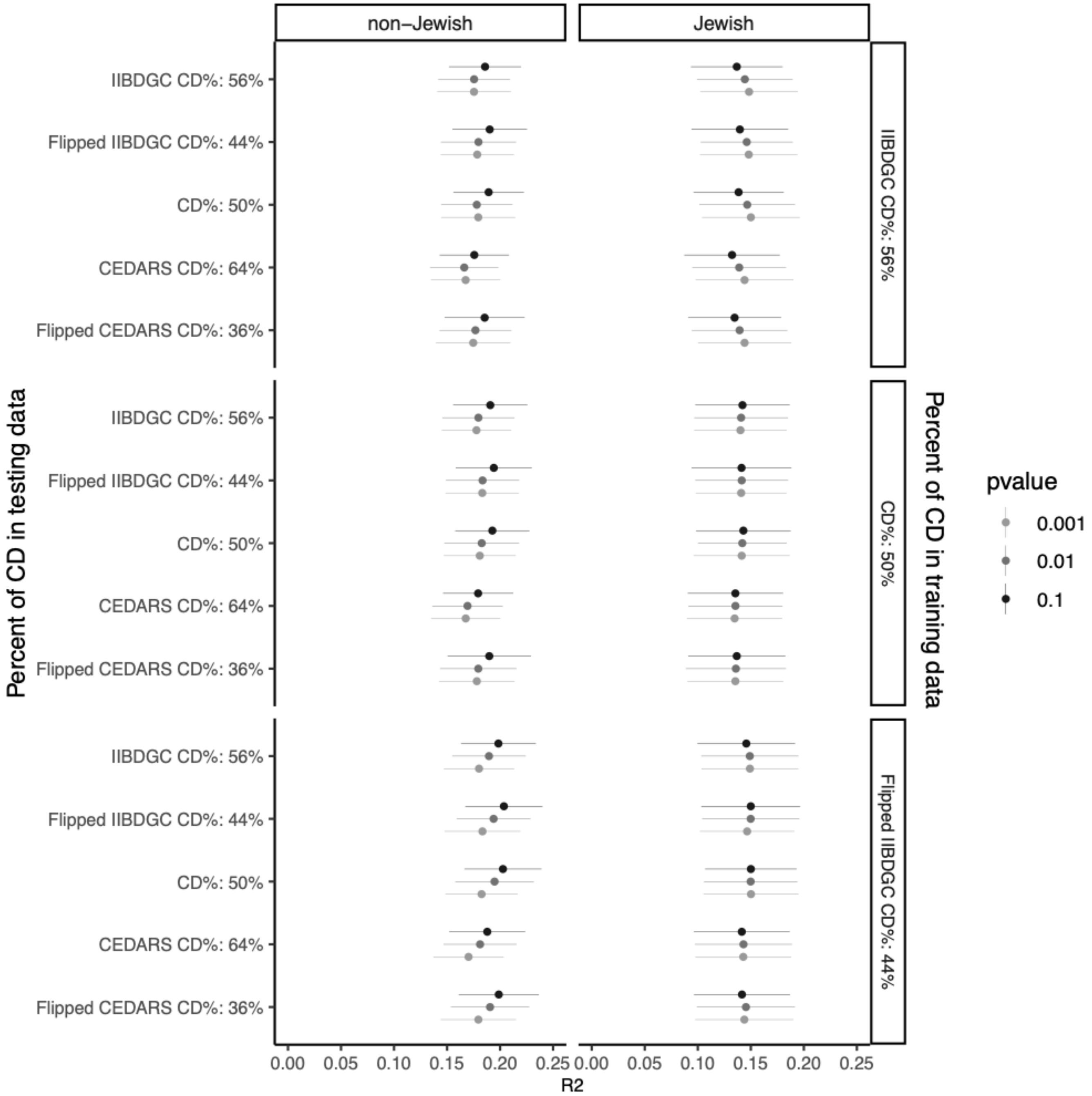

Figure S4. Variance explained by genome-wide prediction models with different CD/UC ratios of training and testing data, Related to Figure 1. The genetic model was trained on IIBDGC with CD/UC ratio (shown in the box on the right): IIBDGC original ratio (CD: 56%, UC: 44%), balanced ratio (CD: 50%, UC: 50%), flipped IIBDGC ratio (CD: 44%, UC: 56%); and tested on CEDARS with CD/UC ratio (shown on the left): IIBDGC original ratio, flipped IIBDGC ratio, balanced ratio, CEDARS original ratio (CD: 64%, UC: 36%), flipped CEDARS ratio (CD: 36%, UC: 64%). Genetic risks were predicted by the P+T method with the P-value cut-off at 0.1, 0.01, and 0.001. Error bar: 95% confidence interval.

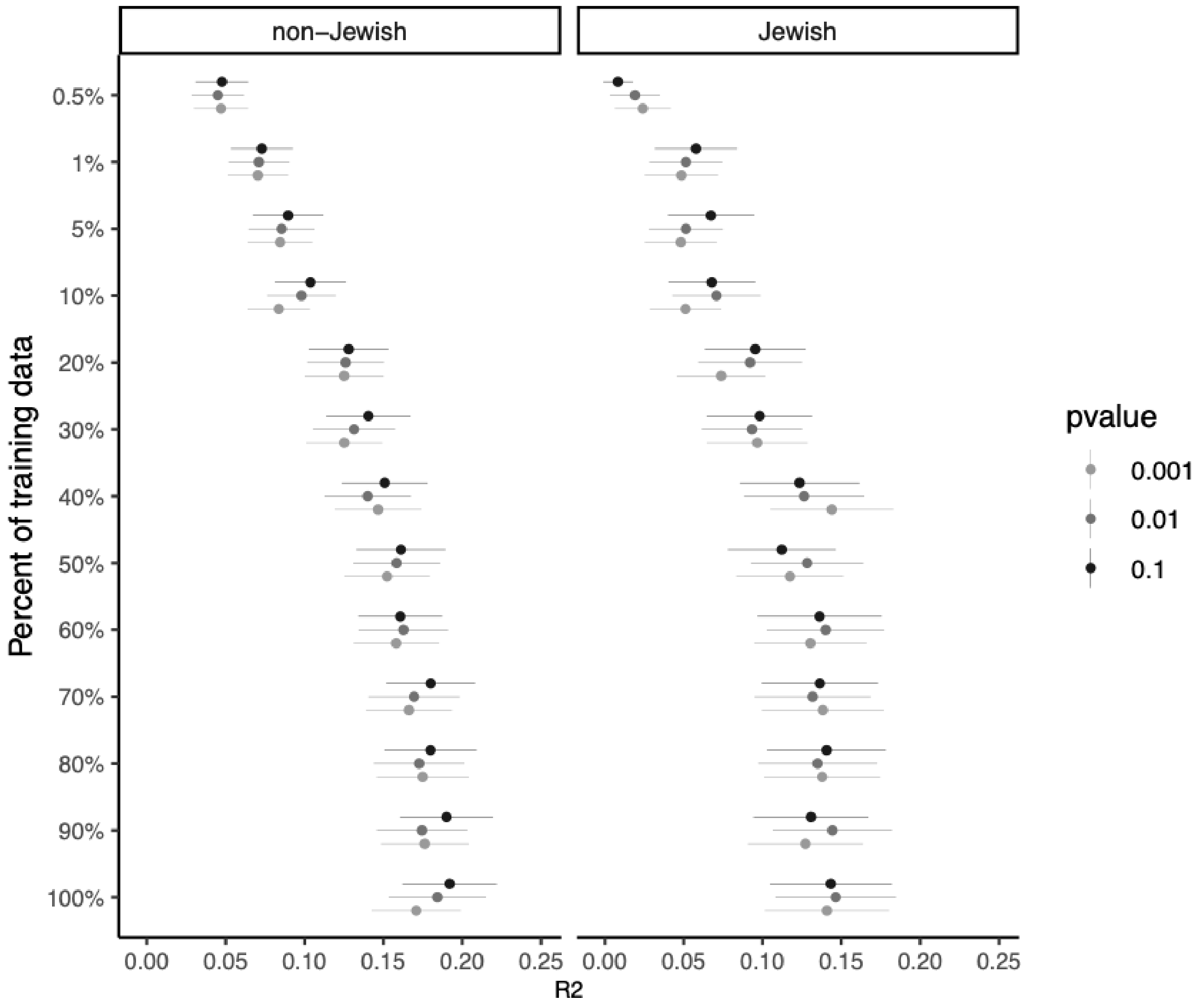

Figure S5. Performance of genetic prediction models trained on various training sample size, Related to Figure 1. The genetic prediction models trained with 0.5% - 100% of IIBDGC samples were tested on non-Jewish (NJ) and Jewish (J) CEDARS samples. The genetic risks were predicted by the P+T method with the P-value cut-off at 0.1, 0.01, and 0.001. Error bar: 95% confidence interval.

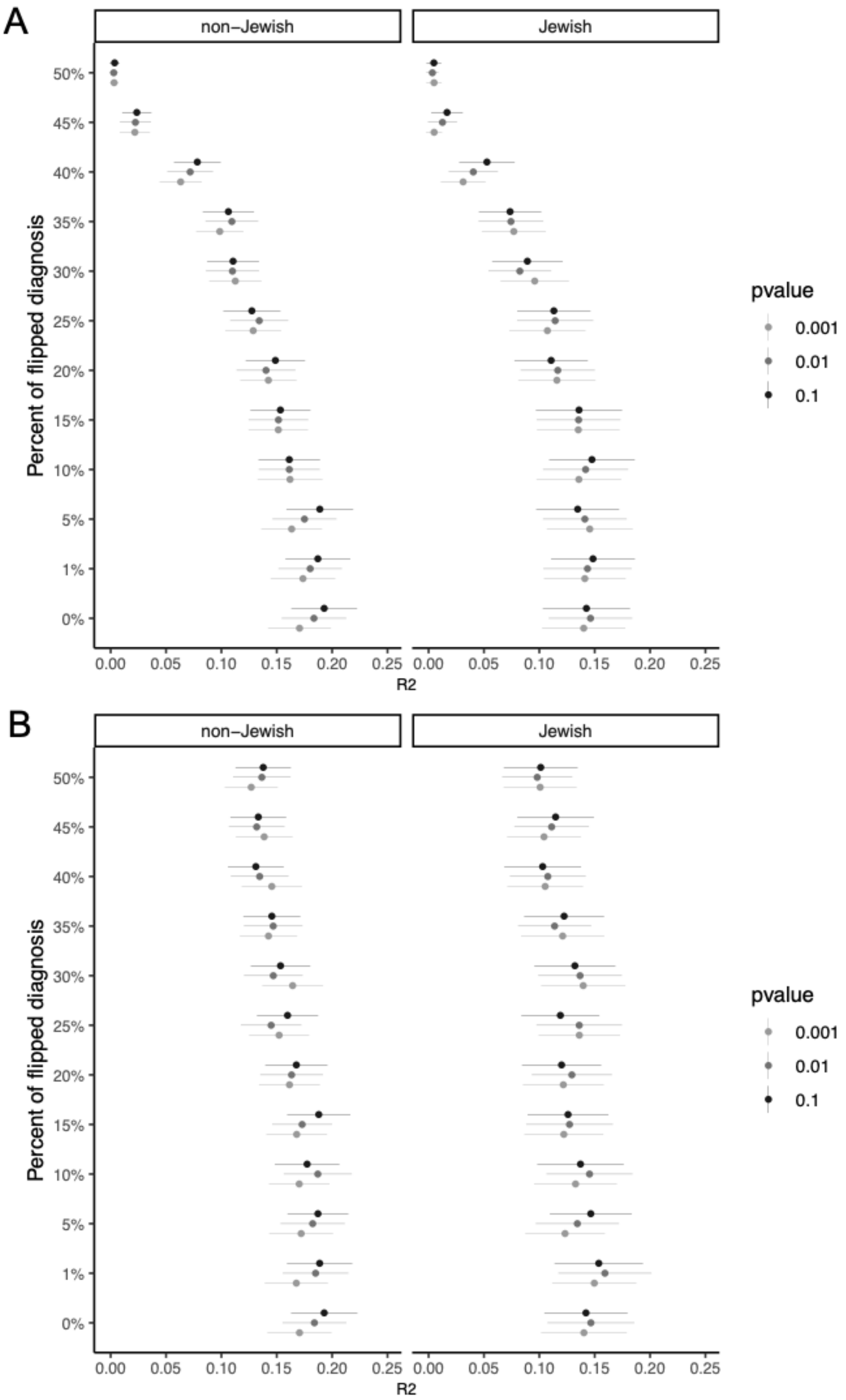

Figure S6. Performance of genetic prediction models trained with a proportion of subjects with their CD:UC diagnosis flipped, Related to Figure 1. The genetic prediction models were trained with the IIBDGC data with a randomly selected proportion of CD and UC subjects flipped to each other to create a "misdiagnosis noise". We randomly selected 0%-50% of total samples for the flipping, and evaluated the performance of our model in non-Jewish and Jewish CEDARS samples respectively. Within the selected samples, we flipped CD and UC (A) in an equal proportion of 1:1; or (B) in a ratio of 9:1 mimicking the diagnosis in clinical practice, e.g., 9 CDs were flipped to UC for 1 UC flipped to CD. Genetic model using the P+T method with P-value cut-off at 0.1, 0.01, and 0.001. Error bar: 95% confidence interval.

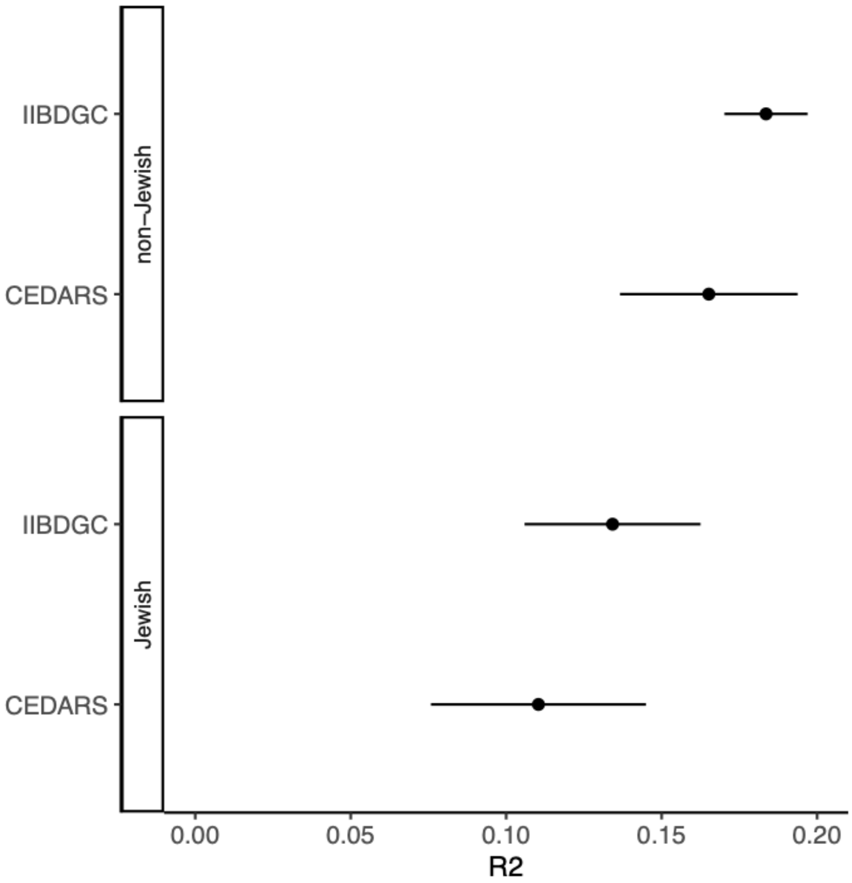

Figure S7. Models tested on IIBDGC and CEDARS samples have similar performance, Related to Figure 1. The genetic model trained using 50% IIBDGC samples were tested on the remaining 50% IIBDGC samples (IIBDGC) and the CEDARS samples (CEDARS). The variance explained by the genetic model are 0.183±0.03 (mean ± 95% confidence interval) and 0.165±0.029 for non-Jewish subjects, and 0.134±0.028 and 0.110±0.035 for Jewish subjects, in IIBDGC and CEDARS, respectively. Error bar: 95% confidence interval.

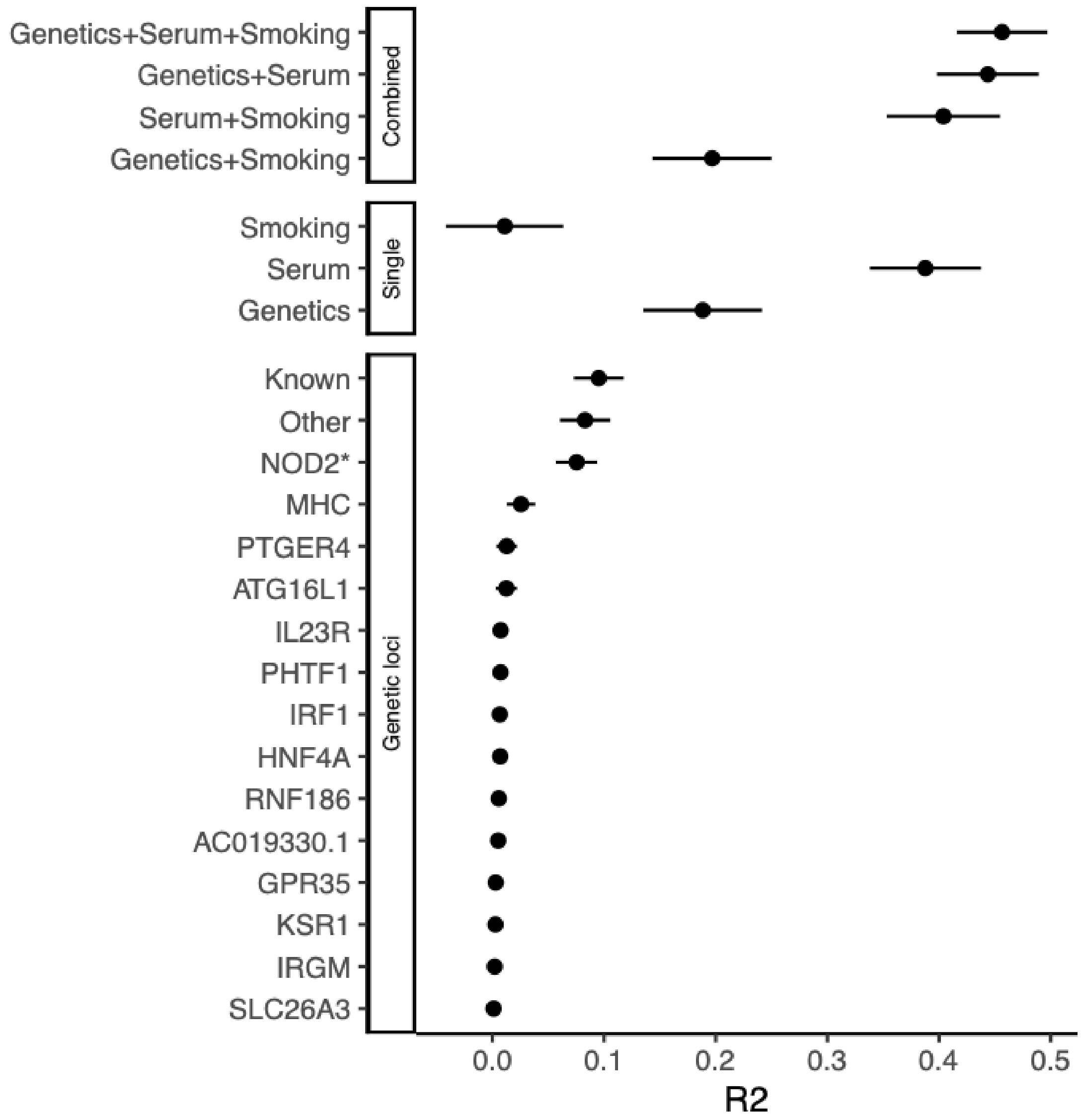

Figure S8. Variance explained by locus-based genetic prediction models, genetic, serum biomarkers, smoking, and their joint models, Related to Figures 1 and 2. *P*-value cut-off of 0.1 was used except for *NOD2* for which the putative causal variants from fine-mapping were used (and therefore marked with ‘*’). Models were tested on non-Jewish and Jewish CEDARS samples. Known: models using index variants from known IBD loci; Other: models using genomic regions other than regions listed in the figure. Error bar: 95% confidence interval.

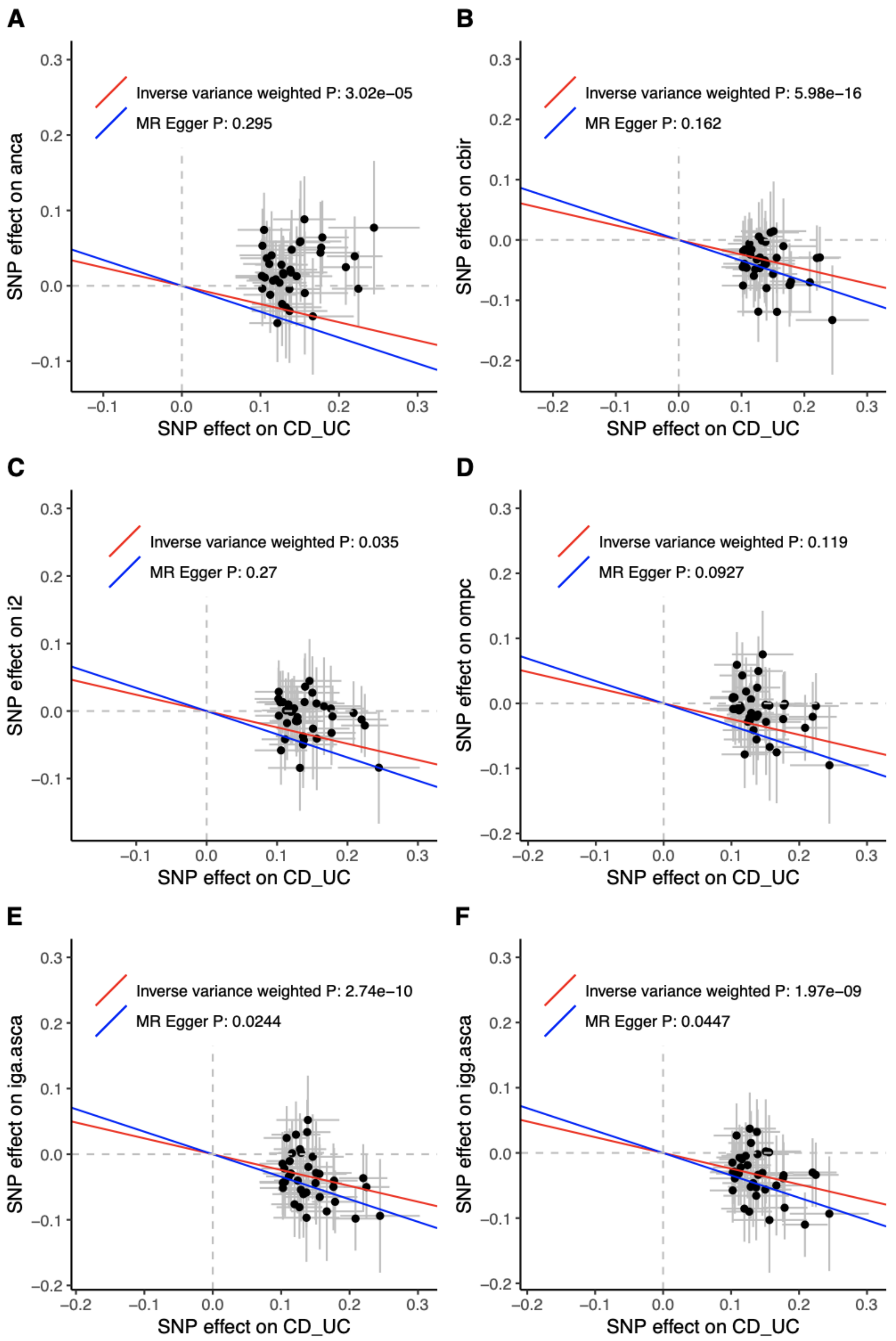

Figure S9. MR analyses for the causal effect of IBD subtype on serum biomarkers, Related to Figure 2. The MR analyses were conducted by using the Inverse variance weighted (red line) and MR-Egger (blue line) method. The slope of the line indicated the estimated causal effect. P: P-value of the causal effect from each method. A. ANCA. B. CBir1. C.I2. D. OmpC. E. ASCA.IgA. F. ASCA.IgG. Error bar: 95% confidence interval.

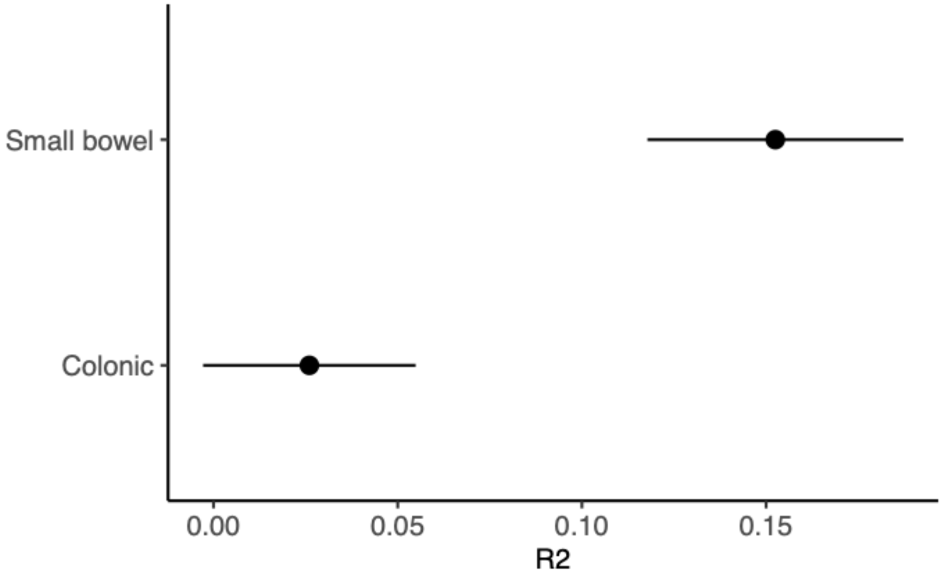

Figure S10. The genetic model has reduced accuracy for colonic CD vs. UC, Related to Figure 3. For a fair comparison, we sampled the IIBDGC non-Jewish subjects such that the training data has the same number of colonic CD and small bowel CD: 2,737 colonic CD, 2,737 small bowel CD and 2,700 UC. The testing data was sampled from CEDARS with 276 colonic CD and 276 UC for testing colonic CD vs. UC, and 999 small bowel CD and 999 UC for predicting small bowel CD vs. UC. The analysis was repeated 1,000 times to evaluate the variance. Error bar: 95% confidence interval.

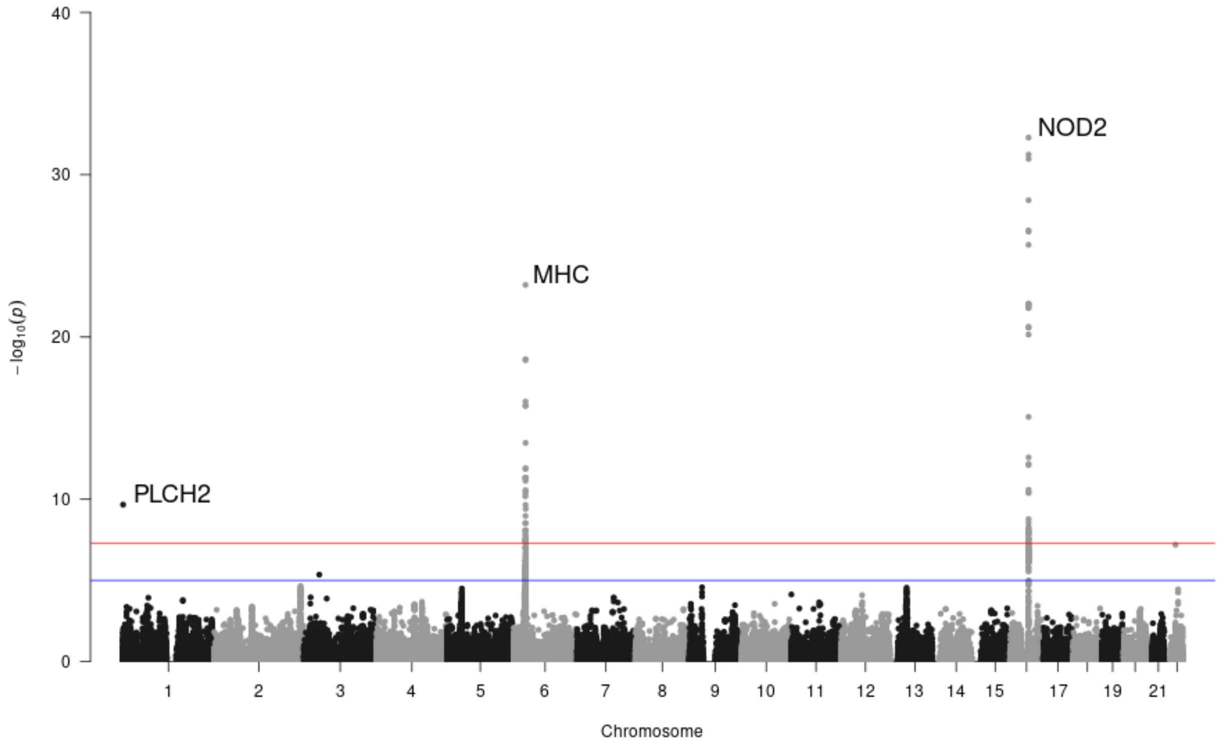

Figure S11. Genetic association analysis for colonic CD versus small bowel CD using IIBDGC non-Jewish samples, Related to Figure 3. Manhattan plot with top associations annotated with a gene within ±300kb. Red line: Genome-wide significance threshold at $P=5\times10^{-8}$. Blue line: suggestive significance threshold at $P=10^{-5}$.

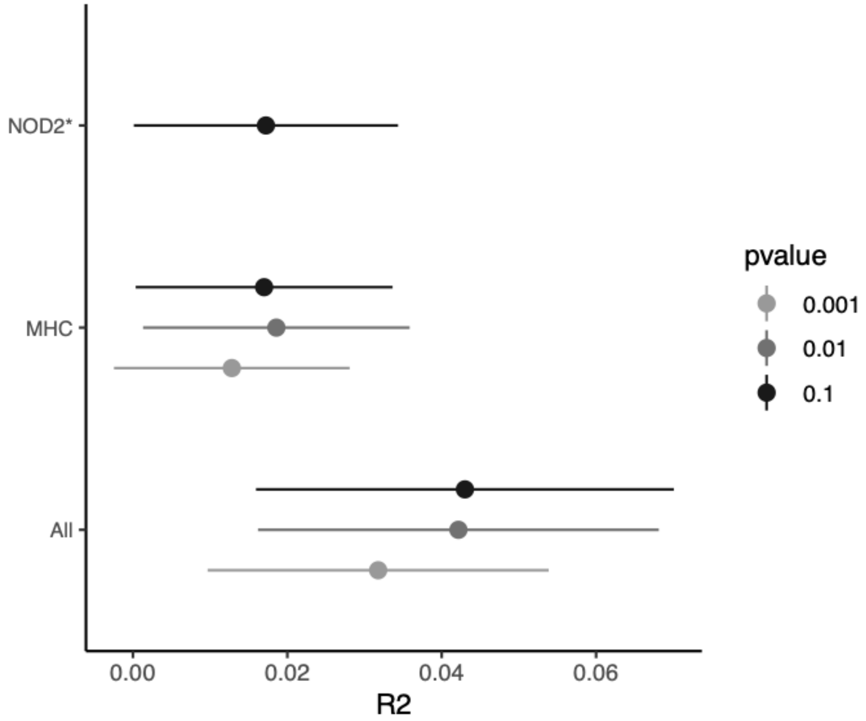

Figure S12. Variance explained by genome-wide or locus-based genetic prediction models on CD location (colonic CD vs. small bowel CD), Related to Figure 3. Models were trained on IIBDGC non-Jewish CD subjects and tested on CEDARS non-Jewish CD subjects. *P*-value cut-off of 0.1, 0.01, and 0.001 were used respectively to build the models except for *NOD2* for which the putative causal variants from fine-mapping were used (*). All: the genome-wide model; Error bar: 95% confidence.

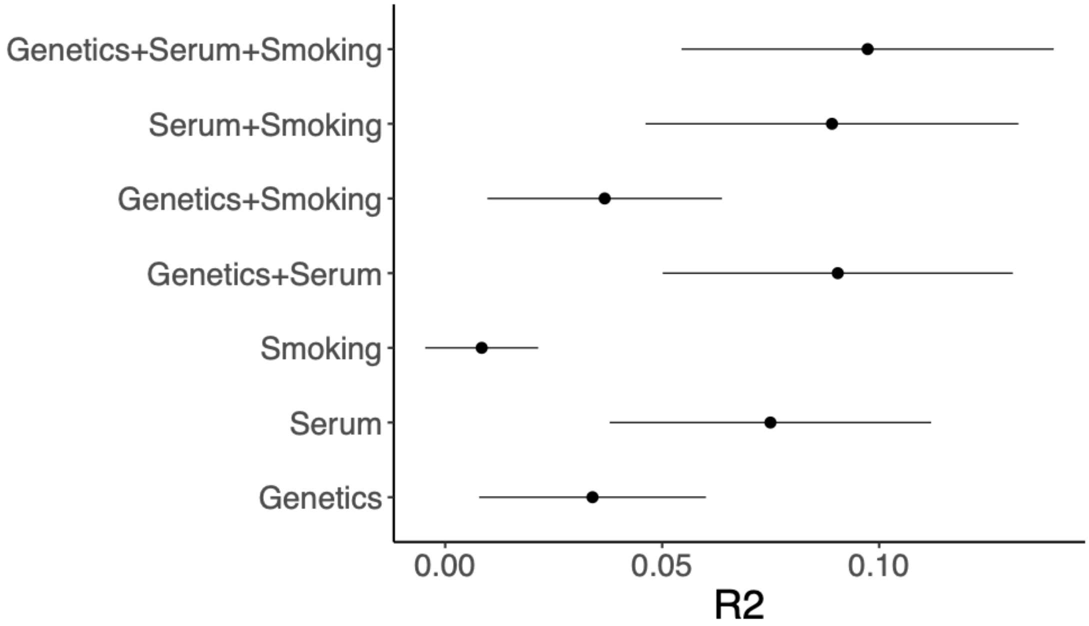

Figure S13. Variance of CD location explained by models with genetics, serum biomarkers, smoking and their combinations, Related to Figure 3. Models were built for the classification of colonic CD and small bowel CD. Error bar: 95% confidence interval.

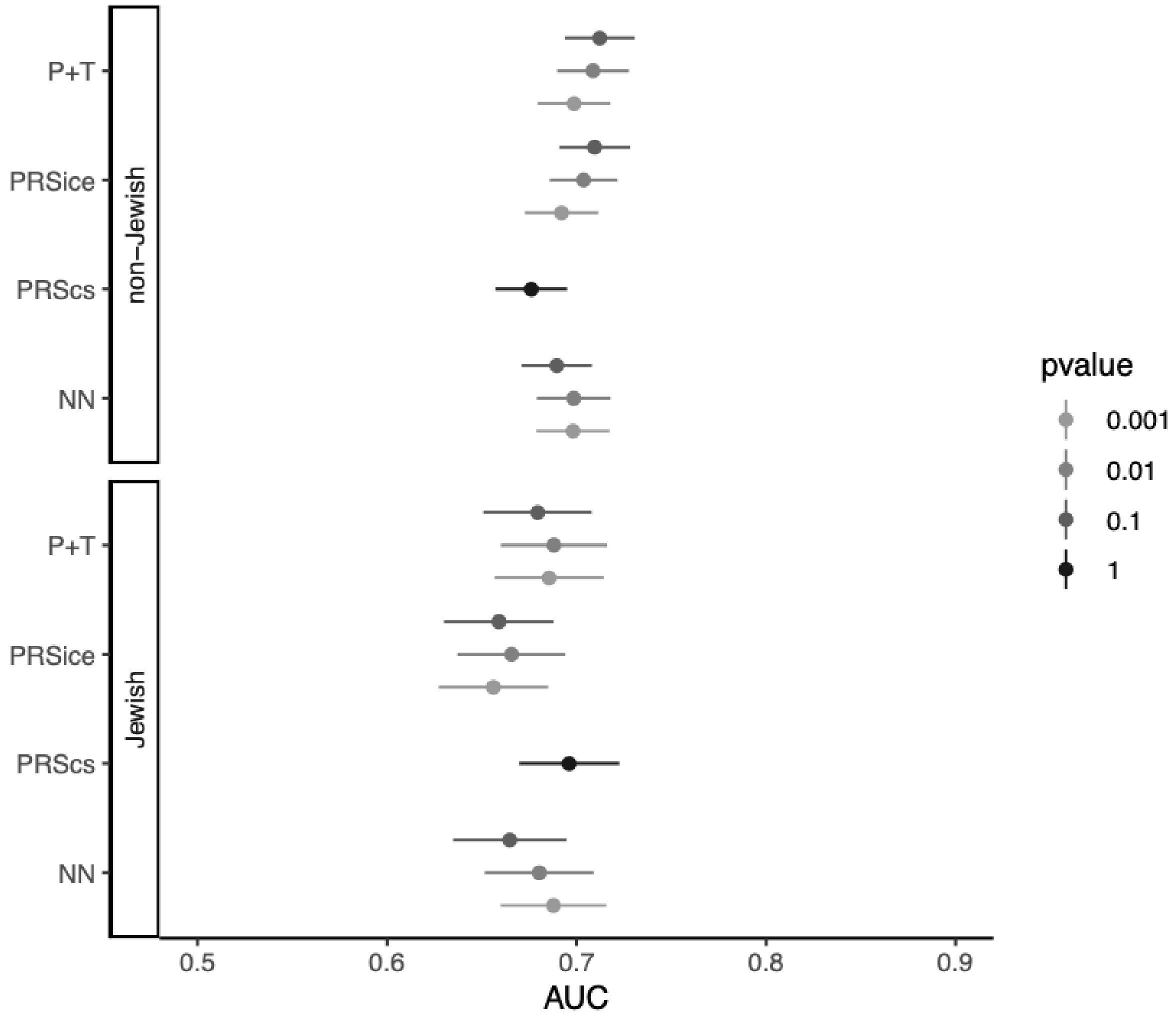

Figure S14. Area under the ROC Curve of the genetic prediction models, Related to Figure 1. The genetic models trained on IIBDGC non-Jewish samples were tested on non-Jewish and Jewish CEDARS samples respectively. The P+T model shows the same performance of prediction as PRSice, PRScs, and the neural network. P+T: genetic risks were predicted by the P+T method with P-value cut-off at 0.1, 0.01, and 0.001. PRSice: genetic model using the PRSice method with P-value cut-off at 0.1, 0.01, and 0.001. PRScs: genetic model using the Bayesian PRS method (P-value = 1: no P-value cutoff). NN: genetic risks were predicted by the multi-layer perceptron neural network with variants from clumping with P-value cut-off at 0.1, 0.01, and 0.001. AUC: Area under the ROC Curve.
